# Robot-Assisted Excision of a Urachal Cyst Causing Dyspareunia and Dysorgasmia: Report of a Case

**DOI:** 10.1089/cren.2015.0029

**Published:** 2016-01-01

**Authors:** Patrick S. Kilday, David S. Finley

**Affiliations:** Department of Urology, Kaiser Permanente Los Angeles, Los Angeles, California.

## Abstract

***Background:*** Urachal remnants are a group of rare anatomical anomalies that include cysts, diverticula, and tumors. We present a case of a young female patient with dyspareunia and dysorgasmia related to a urachal cyst.

***Case:*** A patient with unique presentation of urachal cyst treated robotically. Patient had complete resolution of symptoms postoperatively.

***Conclusion:*** Robot-assisted excision of the urachal remnant provided durable symptom relief.

## Introduction

The true incidence of urachal anomalies is unclear because most urachal remnants remain asymptomatic. While screening ultrasound studies of asymptomatic children have shown urachal remnants to be common, in practice, we only learn about the ones that become symptomatic. When a urachal remnant becomes inflamed or infected, patients will have fever, erythema, pain, hematuria, urinary retention, urinary tract infection, or an abnormal midline mass. Occasionally, patients may present with an acute abdomen or rarely dyspareunia.^1^

Treatment of these anomalies has typically involved removal by open or laparoscopic surgery. In the present case, we describe a young female with a urachal cyst that presented acutely with a surgical abdomen and later caused chronic intermittent lower abdominal pain, dyspareunia, and dysorgasmia. The patient was managed with robotic excision of the cyst and had subsequent resolution of her symptoms.

## Case

A 29-year-old female presented to the emergency department with severe abdominal pain. A CT scan showed a possible urachal diverticulum with signs of surrounding infection and inflammation. Her urine culture grew out *Escherichia coli* and *Enterococcus faecalis*. She defervesced with antibiotics and conservative measures.

**FIG. 1.**
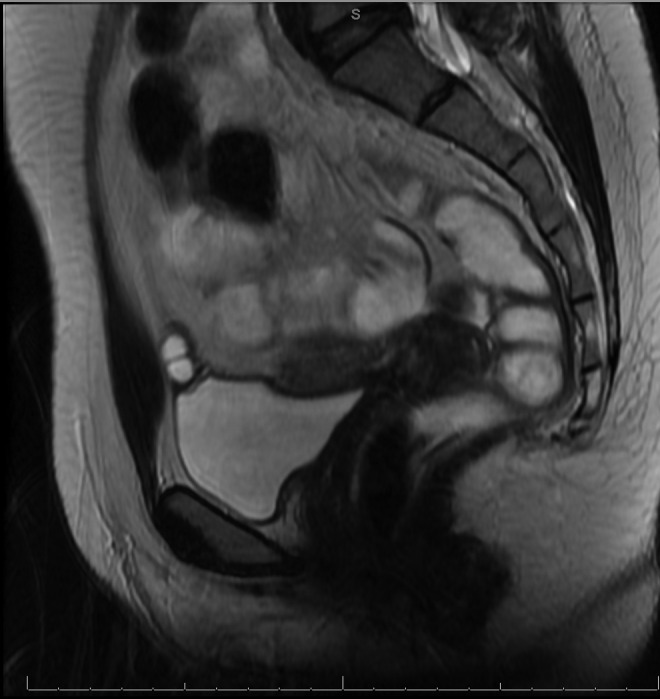
A sagittal view of the urachal cyst and bladder.

**FIG. 2.**
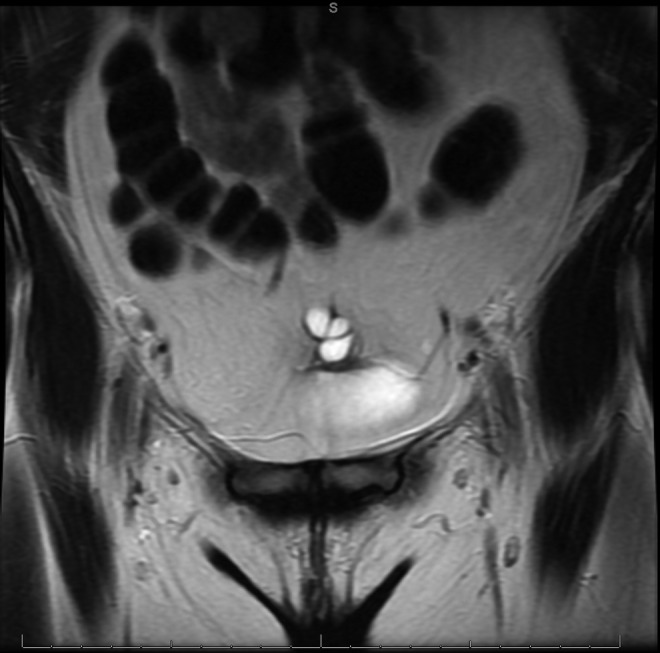
A coronal view showing the cyst and bladder.

Eight months later, she was referred to urology with a history of chronic suprapubic pain, dyspareunia, and dysorgasmia. The pain was described as intermittent lasting minutes to hours. She also noted soreness in the suprapubic area and bloating. Follow-up MRI revealed a benign-appearing multilocular urachal cyst with thin nonenhancing septations as depicted in Figures 1 and 2. On cystoscopy, the bladder appeared normal. However, palpation of the tender abdominal area correlated cystoscopically with the dome of the bladder in the region of the urachal remnant. She opted for cyst removal in an attempt to alleviate her symptoms and reduce the risk of reinfection or malignant transformation.

**FIG. 3.**
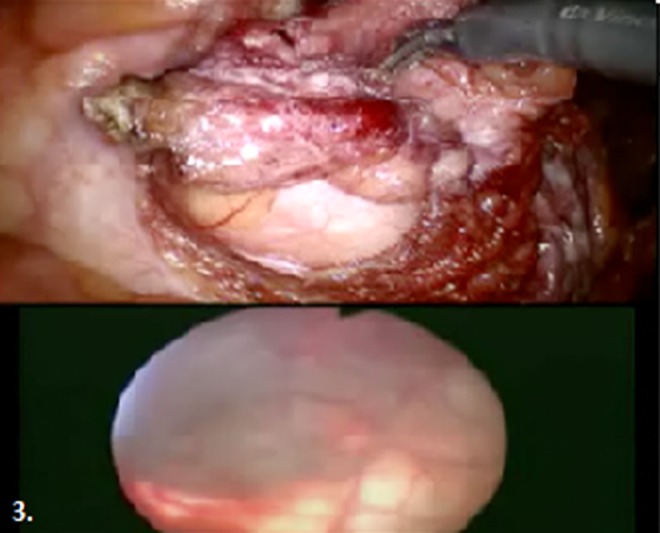
Surgeon's view with simultaneous endoscopic (*upper*) and cystoscopic (*lower*) images to better delineate the cyst and bladder borders.

The patient was positioned in the steep Trendelenburg position and an 18F three-way Foley catheter was inserted. We insufflated the abdomen supraumbilically using a Veress needle. The procedure was done transperitoneally with port placement similar to a standard robotic prostatectomy. The urachus was divided and the bladder was mobilized from the anterior abdominal wall by incising the peritoneum lateral to the medial umbilical ligaments. The space of Retzius was developed. A flexible cystoscope was placed into the bladder to illuminate the bladder dome and urachal remnant—this video feed was transmitted into the Tile Pro™ software of the robot to allow simultaneous cystoscopic visualization through the robotic console illustrated in Figure 3. The urachal cyst was mobilized and skeletonized down to a small neck at its insertion. The detrusor muscle layer was incised with thermal energy just below the neck of the cyst but superficial to the bladder mucosa. The cyst was extracted and sent for frozen section, which was negative for malignancy, obviating the need for partial cystectomy. The overlying detrusor was repaired with a 3–0 V-Loc followed by a second 3–0 V-Loc on the muscle and peritoneum. The bladder was filled and the repair was noted to be watertight.

The patient was discharged home the same day. Her catheter was removed on day 3. She has remained symptom free over the past 12 months since surgery.

## Discussion

Embryologically, the urachus is the remnant of the allantois, which connects the urogenital sinus (the upper portion forms the bladder) with the umbilicus. During the fifth month of development, as the bladder descends into the pelvis, it pulls the urachus down with it creating a canal. Then, the urachus normally obliterates, forming the median umbilical ligament, which lies between the transversalis fascia and the peritoneum.^2^

When the urachal channel persists, it can lead to a spectrum of anomalies ranging from a cyst to a sinus diverticulum or a fistulous tract. Most urachal remnants are asymptomatic and they account for about 3 in 200,000 hospital admissions. Patients usually present between the ages of 16 and 35 years.^2^ Common symptoms include clear umbilical drainage, fevers, and pain. These patients may also be picked up with an abnormal physical examination or radiologic finding. Rarely will patients develop a malignant transformation of the urachal remnant. In these rare instances, it is typically adenocarcinoma of the urachus and carries a poor prognosis.

The management is tailored according to the severity of the case. In the current case, the cyst probably became infected imitating an acute abdominal picture. After resolution of the infection, she suffered from dyspareunia and dysorgasmia likely due to smoldering chronic inflammation of the cyst. Dyspareunia appears to be a rare symptom of a urachal cyst. To our knowledge, ours is the second such report. Zanghi and colleagues discussed a 28-year-old woman with a urachal cyst causing dyspareunia and a sensation of fluid flowing in her abdomen.^1^ While the exact link between the urachal cyst and these symptoms is unknown, it is our theory that the increased abdominal pressure associated with orgasm and intercourse may lead to irritation of the chronically infected cyst. This irritation may manifest as pain and may help explain why excision of the mass led to the immediate relief of her symptoms. The constellation of symptoms in the report by Zanghi and colleagues closely mirrors those of our patient.

Symptomatic urachal cysts may initially be treated conservatively with antibiotics and require percutaneous drainage, however, they will often recur. Therefore, if symptoms persist or if malignancy is suspected, surgical excision is recommended. Open, laparoscopic, and robotic approaches have been effectively utilized. While most of the minimally invasive studies discuss a laparoscopic approach to the urachal remnant, recently, several case reports have been published using a robotic approach for the treatment of urachal remnants.^3,4^ The robotic approach is unanimously considered to improve cosmesis and visualization compared with open surgery. In our case, the 3D vision and the dexterity of the robot significantly aided with the dissection of the urachal cyst and bladder reconstruction. We found that the use of a flexible cystoscope concurrently with robotic dissection of the cyst provided a valuable extra layer of real-time information; this allowed us to fully excise the cyst without having a cystotomy. Real-time cystoscopic assistance would have been particularly helpful if the mass involved the mucosa or exhibited malignant features as this would have necessitated a partial cystectomy.

There are inherent limitations to a case report. Our case illustrates effective robotic excision of a urachal cyst with complete symptom resolution. However, pain or dyspareunia alone may not be sufficient cause to warrant the risks of surgery because of the complex and often multifactorial nature of this entity. Due to the wide range of symptoms that urachal anomalies may cause, one must consider the entire clinical scenario. We therefore cannot be certain that future patients would have similar results even with similar symptomatology.

## Conclusion

This case represents a unique presentation of a chronically inflamed/infected urachal cyst with intermittent dyspareunia and dysorgasmia. The cyst was effectively treated with robotic excision and the patient had subsequent relief of her symptoms. We believe that robotic excision of urachal remnants offers similar benefits to those afforded by laparoscopic surgery, with added dexterity and visualization. The optimal management of this rare malady remains to be determined.

## Abbreviations Used

CT: computed tomography
MRI: magnetic resonance imaging
